# Antibacterial Vancomycin@ZIF-8 Loaded PVA Nanofiber Membrane for Infected Bone Repair

**DOI:** 10.3390/ijms23105629

**Published:** 2022-05-18

**Authors:** Yunbo Zhao, Hongshui Wang, Xianrui Zou, Donghui Wang, Ying Fan, Xiaoyan Zhao, Mingjun Li, Lei Yang, Chunyong Liang

**Affiliations:** 1 Tianjin Key Laboratory of Materials Laminating Fabrication and Interface Control Technology, School of Materials Science and Engineering, Hebei University of Technology, Tianjin 300130, China; 15733153963@163.com (Y.Z.); kingflood@hebut.edu.cn (H.W.); zouxianrui@163.com (X.Z.); wdh_81@163.com (D.W.); fanying_99@yahoo.com (Y.F.); 2 School of Civil and Transportation Engineering, Hebei University of Technology, Tianjin 300130, China; zhaoxiaoyan@hebut.edu.cn; 3 Center for Health Science and Engineering, School of Health Sciences and Biomedical Engineering, Hebei University of Technology, Tianjin 300130, China; ylei@hebut.edu.cn; 4 Fujian Provincial Key Laboratory for Advanced Micro-Nano Photonics Technology and Devices, Research Center for Photonics Technology, Quanzhou Normal University, Quanzhou 362000, China

**Keywords:** ZIF-8, drug loading, electrospinning, antibacterial activity, cell adhesion

## Abstract

Bone substitutes with strong antibacterial properties and bone regeneration effects have an inherent potential in the treatment of severe bone tissue infections, such as osteomyelitis. In this study, vancomycin (Van) was loaded into zeolitic imidazolate framework-8 (ZIF-8) to prepare composite particles, which is abbreviated as V@Z. As a pH-responsive particle, ZIF-8 can be cleaved in the weak acid environment caused by bacterial infection to realize the effective release of drugs. Then, V@Z was loaded into polyvinyl alcohol (PVA) fiber by electrospinning to prepare PVA/V@Z composite bone filler. The drug-loading rate of V@Z was about 6.735%. The membranes exhibited super hydrophilicity, water absorption and pH-controlled Van release behavior. The properties of anti *E. coli* and *S. aureus* were studied under the pH conditions of normal physiological tissues and infected tissues (pH 7.4 and pH 6.5, respectively). It was found that the material had good surface antibacterial adhesion and antibacterial property. The PVA/V@Z membrane had the more prominent bacteria-killing effect compared with the same amount of single antibacterial agent containing membrane such as ZIF-8 or Van loaded PVA, and the antibacterial rate was up to 99%. The electrospun membrane had good biocompatibility and can promote MC3T3-E1 cell spreading on it.

## 1. Introduction

Severe bone tissue infections, such as osteomyelitis, have always been a difficult problem in orthopedic treatment [1]. It refers to the bone defect accompanied by bacterial infection. The main clinical treatment method is to remove the infected tissues and give strict antibiotic treatment [2]. This method has a long operation cycle and will bring great pain to patients. In addition, the use of systemic antibiotics makes the target site lack sufficient antibiotics, which is often accompanied by toxic side effects and bacterial drug resistance [3,4]. Therefore, it is necessary to design a composite bone filling material that can achieve efficient drug release at the target location and promote bone tissue regeneration [5].

Metal organic frameworks (MOFs) are the porous material composed of metal ions and organic ligands. They have been widely used in biomedical materials because of their good biocompatibility, high porosity and chemical stability [6,7,8]. Among a variety of MOFs, zeolitic imidazolate framework-8 (ZIF-8) is a pH-responsive particle and can be cleaved in the micro acid environment of bacterial infection, which improves the effectiveness of drug loading and reduces adverse effects [9,10]. ZIF-8 also has a good application prospect in bone tissue regeneration. Zn can regulate the secretion and expression of osteogenic-related gene, and it also has anti-inflammatory and antibacterial effects to solve the problem of implant infection [11,12].

Vancomycin (Van), a glycopeptide antibiotic, has traditionally been used as a “last-line drug” to treat serious infections where many antibiotics are ineffective [13,14]. Van plays a bactericidal role by blocking the synthesis of high molecular peptidoglycan constituting the bacterial cell wall, and it has low cytotoxicity and will not affect the osteogenic differentiation of cell [15]. Van is a heat-stable antibiotic, which can withstand high temperature of 121 °C for 15 min [16,17]. Researchers have successfully encapsulated Van in ZIF-8 particles and modified the surface with hyaluronic acid (HA). The particles present biosafety and pH controlled release behavior, and they can target bacterial infection sites to achieve effective sterilization [14]. Polyvinyl alcohol (PVA) was used as a substrate in this work. Compared with other polymers that must be dissolved in organic solvents, PVA effectively avoids the toxicity caused by organic solvents [18]. Moreover, a number of studies have demonstrated the application of PVA in bone and cartilage tissue engineering scaffolds, which have verified its good biocompatibility [19,20,21].

**Scheme 1:**
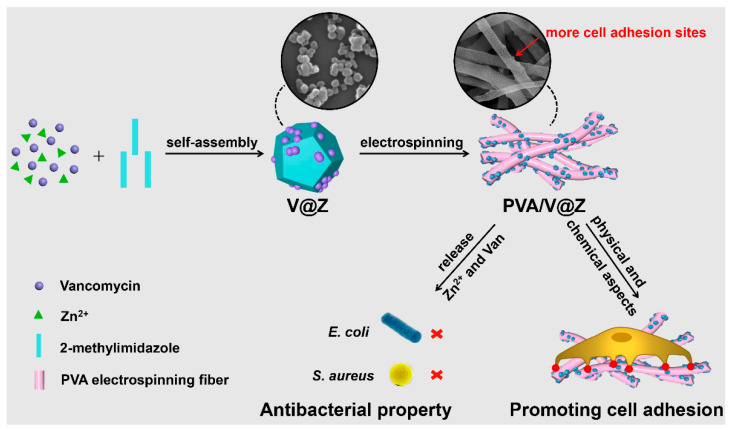
The preparation routes and biological applications of PVA/V@Z membrane.

Here, Van was encapsulated into ZIF-8 to synthesize V@Z nanoparticle, and drug delivery system PVA/V@Z bone filler was prepared by electrospinning, as shown in Scheme 1. The structure of fibers prepared by electrospinning can well simulate the structure of extracellular matrix (ECM). In addition, the Zn element in ZIF-8 is the important element in cell growth [22]. Therefore, the material can promote the growth of cells from both physical and chemical aspects. Under the pH conditions of normal physiological tissue and infected tissue (pH 7.4 and pH 6.5, respectively), the release of Van was investigated. The surface antibacterial adhesion and sustained-release bactericidal properties of the materials were investigated under these two pH values. The effect of electrospun membrane on the toxicity and spreading behavior of MC3T3-E1 cells was also studied.

## 2. Results and Discussion

### 2.1. Materials Characterization

**Figure 1:**
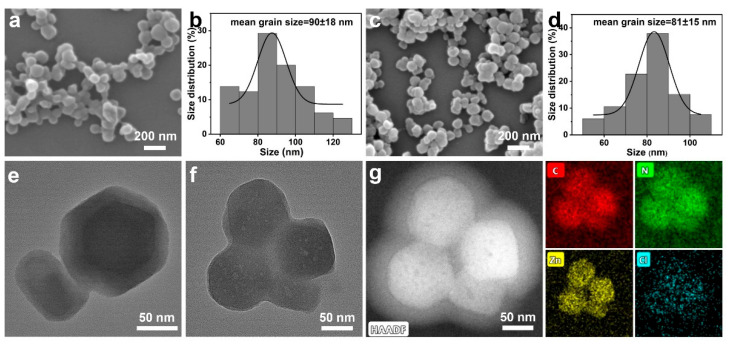
SEM images and size distributions of (**a**,**b**) ZIF-8 and (**c**,**d**) V@Z particles; TEM images of (**e**) ZIF-8 and (**f**) V@Z particles; (**g**) Elemental mapping of V@Z particles.

The self-assembled ZIF-8 and V@Z were designed in this work according to a previous report [11], presenting enhanced antibacterial activity as well as cell-spreading behavior. The morphology of ZIF-8 (Figure 1a,e) and V@Z (Figure 1c,f) nanoparticles were characterized by scanning electron microscopy (SEM) and transmission electron microscopy (TEM). Figure 1b,d show that the particle size distributions of ZIF-8 and V@Z are 90 ± 18 nm and 81 ± 15 nm, respectively. Figure 1g presents the homogenous distribution of relevant C, N, Zn, and Cl elements of V@Z. Cl is a characteristic element in Van, which indicates that Van is successfully encapsulated in V@Z.

The particle composition and drug encapsulation were further characterized by X-ray diffraction (XRD) and Fourier transform infrared spectroscopy (FTIR). The XRD patterns in Figure S1a confirm the characteristic peaks (7.3°, 10.4°, 12.8°, 14.8°, 16.5°, 18.1°, 22.2°, 24.6°, 26.7°, 29.8°) of ZIF-8 and V@Z [23,24], suggesting that the addition of Van did not affect the composition of ZIF-8. Figure S1b shows the characteristic diffraction peaks of ZIF-8 at 3135, 2925, 1584 cm^−^^1^, corresponding to the aromatic and aliphatic C-H groups and the stretching vibrations of C=N in imidazole, respectively. The peaks at 1146 and 995 cm^−^^1^ correspond to the stretching vibration peaks of C-N in the imidazole unit [11,25]. The characteristic peaks of Van are located at the hydroxyl stretching at 3274 cm^−^^1^, C=O stretching at 1645 cm^−^^1^ and phenolic hydroxyl at 1227 cm^−^^1^, respectively [14]. The infrared spectra of V@Z particles show these three characteristic peaks of Van, which indicates the encapsulation of Van.

**Figure 2:**
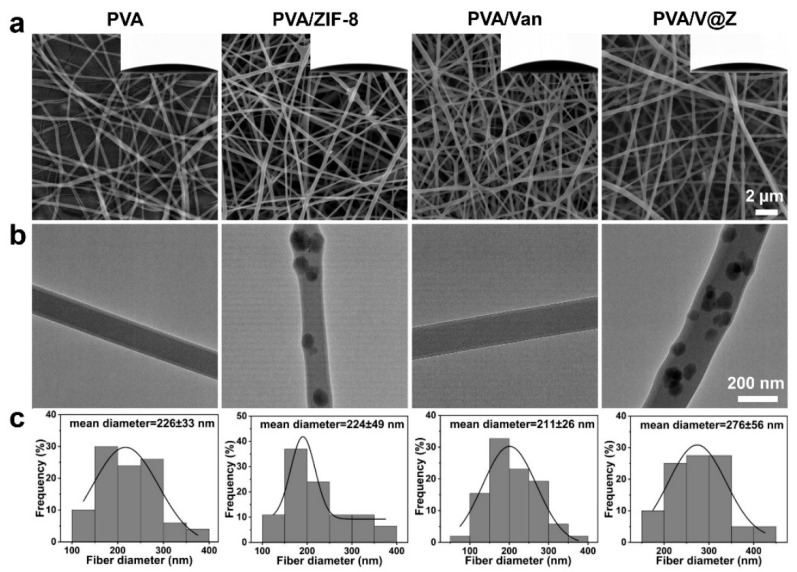
SEM images and contact angle (the insert figure) (**a**), TEM images (**b**), and the diameter distribution (**c**) of different membranes.

PVA is proved to be a biocompatible polymer, which can be applied as an ideal drug delivery system. ZIF-8 and V@Z were encapsulated in PVA electrospun fibers to biocompatibility. In addition, the releasing rates of Zn and Van were lower compared with pure ZIF-8 or V@Z, which prolonged the working life. The structure and fiber morphology of membranes include PVA, PVA/ZIF-8, PVA/Van and PVA/V@Z, as shown in Figure 2. The high porosity and interconnected fiber structures can be clearly observed from the SEM images (Figure 2a). The water contact angles show that the four membranes maintain good hydrophilicity (the insert figure in Figure 2a). Figure 2c shows the diameter distribution of membranes. It can be seen that the average diameter of PVA fiber is 226 ± 33 nm. After adding ZIF-8, the fiber diameter (224 ± 49 nm) almost remains unchanged. After Van is added, the diameter of fibers decreases slightly to 211 ± 26 nm. After adding V@Z, the fiber diameter increases to 276 ± 56 nm. The change of fiber diameter may be caused by the change of electrical conductivity of electrospinning solution [26].

TEM (Figure 2b) shows that ZIF-8 and V@Z particles are successfully encapsulated into PVA fibers, and the fiber surface became rough compared to PVA and PVA/Van fibers. Figure S2 presents the distribution of relevant C, N, Zn, and Cl elements of PVA/V@Z. It can be seen that N, Zn and Cl elements are mainly concentrated in the distribution area of V@Z particles. This illustrates the successful retention of V@Z in electrospun fibers.

**Figure 3:**
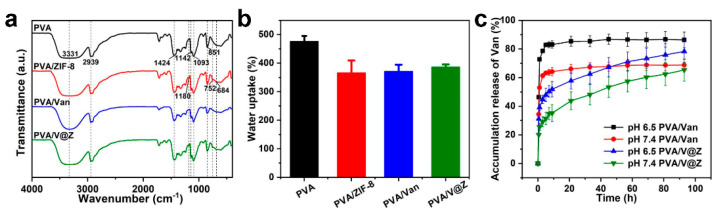
FTIR (**a**) and water absorption (**b**) of different membranes; in vitro releasing profile of Van (**c**) in PVA/Van and PVA/V@Z membranes.

The chemical structures in the membrane were analyzed by FTIR, as shown in Figure 3a. In the FTIR analysis of PVA, the characteristic peaks at 3331, 2939, and 1093 cm^−^^1^ correspond to the stretching vibration peak of -OH, C-H and C-O bond. The characteristic peak at 1424 cm^−^^1^ corresponds to the bending vibration peak of -CH_2_, and 1142 and 851 cm^−^^1^ are the characteristic peaks caused by C-C stretching vibration [27,28]. It can be seen that the characteristic peak of Van cannot be observed in PVA/Van, which may be due to the successful encapsulation of Van into the interior of fibers [29]. In the infrared peak of PVA/ZIF-8 and PVA/V@Z membranes, the characteristic absorption peaks of ZIF-8 at 1180, 752 and 684 cm^−^^1^ can be observed, indicating the encapsulation of ZIF-8 and V@Z particles in them.

Figure 3b shows the different water absorption of the four membranes. PVA contains numerous hydrophilic hydroxyl groups, which gives it good water solubility [30], with a water absorption rate up to 470%. After adding hydrophobic ZIF-8 and V@Z particles, the water absorption rates of the membranes decrease. However, the water absorption of the four membranes is maintained at a high level, which is conducive to the adhesion and proliferation of cells [31]. After calculation, the drug-loading rate of V@Z particles is 6.735 ± 1.5%. Figure 3c is the releasing curve of Van in different electrospun membranes. It shows that the release of Van is fast in the PVA/Van membrane, reaching 86% at pH 6.5 and 68% at pH 7.4 within 10 h. This is because Van is a water-soluble material and will be rapidly released without the ZIF-8 package in PBS. However, the release curve of Van in PVA/V@Z membrane is different; Van can be released slowly and continuously for at least 93 h. At this time, the release rates of Van at pH 6.5 and 7.4 were about 78% and 65%, respectively. This is because ZIF-8 particles can effectively wrap Van and delay its release. Due to the pH responsiveness of ZIF-8, Van is released faster at pH 6.5. This enables the membrane to achieve a more effective antibacterial effect in the slightly acidic environment of bacterial infection.

### 2.2. Antibacterial Properties

**Figure 4:**
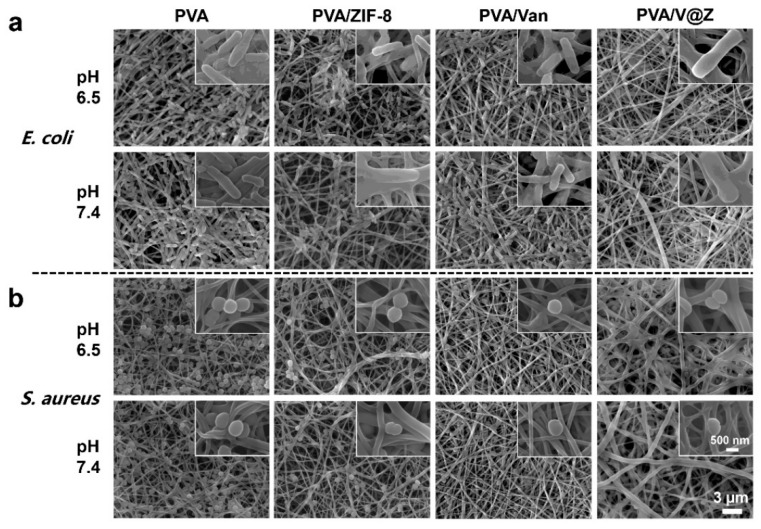
SEM images of different membranes after 12 h co-culture with *E. coli* (**a**) and *S. aureus* (**b**) at pH 6.5 and pH 7.4.

Along with the increasing population of biomedical implants, the complication caused by bacterial infection after the surgery is a global challenge. Once the biofilm is formed on the surface of the implanted materials, it is very difficult to eliminate the biofilms and usually needs to exchange the medical devices. Bacterial adhesion is considered as the key to the formation of biofilms on a polymer membrane [32]. How to avoid the biofilm formation is the key to antibacterial infection. The bacterial adhesion profile on the surface of different membranes was tested after 12 h co-culture with Escherichia coli (*E. coli*) and Staphylococcus aureus (*S. aureus*) at pH 6.5 and 7.4, respectively. Obviously, the surface of PVA is covered with a large number of bacteria (Figure 4). The PVA/ZIF-8 and PVA/Van electrospun membranes have antibacterial effect. Both Zn ions and Van in the membrane can release to the surrounding environment and destroy the structure of the bacterial cell wall, so that bacteria will be killed before adhering to the membrane surface. Therefore, the number of bacteria adhering to the material surface in the composite groups is significantly reduced. Compared with the membrane using a single antibacterial agent, the PVA/V@Z membrane has the best antibacterial effect at pH 6.5 and 7.4. This result shows that the PVA/V@Z electrospun membrane can effectively avoid the formation of bacterial biofilm in both normal and bacterial infected tissue environments.

**Figure 5:**
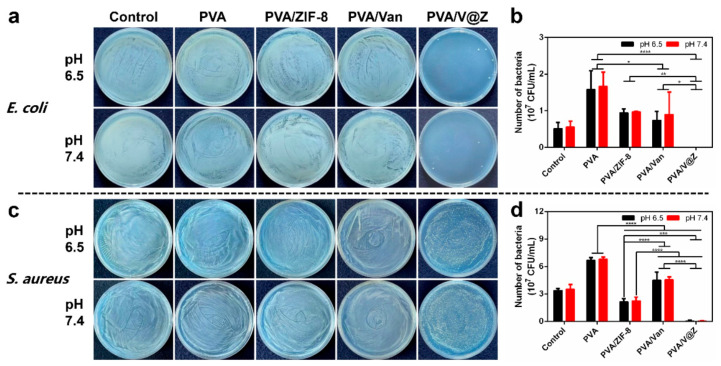
Representative images of agar plates and corresponding data statistics after 24 h co-culture with different membranes: *E. coli* (**a**,**b**) and *S. aureus* (**c**,**d**) (n = 3). (* *p* < 0.05, ** *p* < 0.01, *** *p* < 0.001, **** *p* < 0.0001).

In addition to the adhesion resistance on the surface of the electrospun membrane, its antibacterial property in *E. coli* and *S. aureus* suspensions was also studied. Figure 5 shows the plate obtained by coating the original solution; the number of bacterial colonies in Control, PVA, PVA/ZIF-8 and PVA/Van groups exceeds the quantifiable number, indicating that neither ZIF-8 nor Van alone can effectively reduce the growth of bacteria. However, when ZIF-8 combines with Van to form V@Z, it shows excellent antibacterial properties. We diluted the bacterial colony gradient 10,000 times (Figure S3) and counted the number of bacterial colonies. The data statistics (Figure 5b,d) show that the number of bacterial colonies in the PVA group was significantly higher than that in the control group. This is because the presence of PVA increased the specific surface area in the bacterial growth environment and promoted the adhesion of bacteria. PVA/ZIF-8 and PVA/Van groups also have a certain degree of antibacterial effect. It is worth mentioning that under these two pH values, the antibacterial rate of the PVA/ZIF-8 group against *E. coli* is about 40%, and the antibacterial rate against *S. aureus* is about 70%. The antibacterial effect of ZIF-8 against *S. aureus* is stronger than that of *E. coli*. This is because the cell wall of Gram-negative bacteria is relatively complex. In addition to the lipopolysaccharide and peptidoglycan layer, its surface is also covered with an outer membrane, which can effectively reduce the toxic effect of Zn^2+^ on bacterial cells [33,34]. The antibacterial effect of the PVA/V@Z group on the two bacteria is up to 99%, indicating that the PVA/V@Z group has the best antibacterial effect and could effectively inhibit the growth of bacteria in both normal and bacterial infected tissue environments.

### 2.3. Cell Studies

**Figure 6:**
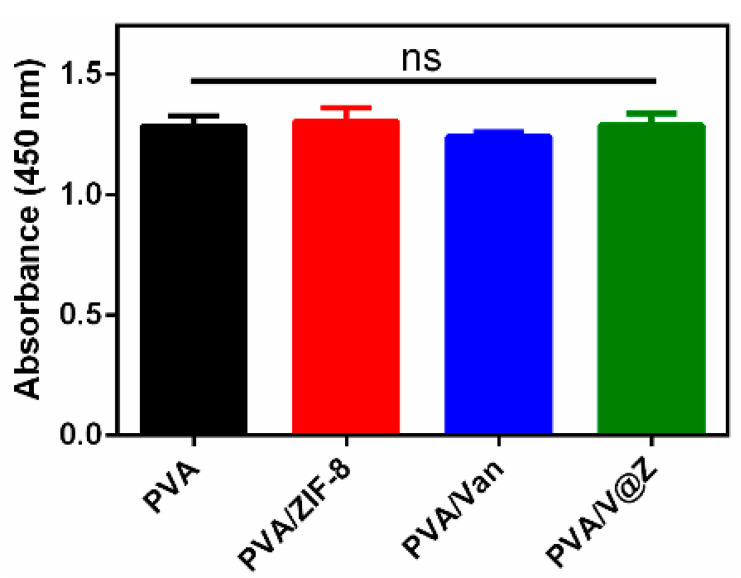
CCK-8 results of MC3T3-E1 cells after 24 h co-culture with different membranes (n = 5). (ns, not significant).

The cell viability after co-culture with different fibrous membrane extracts for 24 h was tested via a CCK-8 assay kit (Figure 6). There is no significant difference between all experimental groups, indicating that the addition of antibiotics will not present toxic to MC3T3-E1 cells. The PVA/V@Z membrane maintains good biocompatibility.

**Figure 7:**
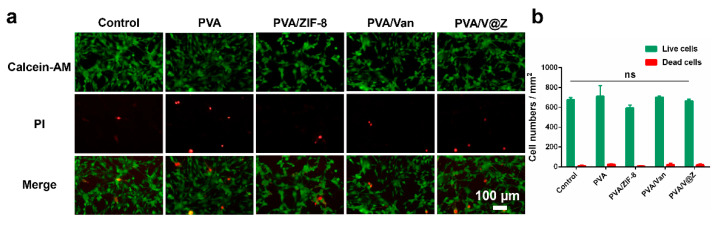
(**a**) Images of live/dead staining of MC3T3-E1 cells after being co-cultured with different fibrous membrane extracts for 24 h. Calcein–AM (green): live cells. PI (red): dead cells; Merge: merged channel of live/dead cells. (**b**) Statistical quantification of the cell number, n is the number of confocal pictures analyzed (n = 5). (ns, not significant).

In order to further explore the cytotoxicity between different membranes, the extract solution of different membranes was prepared for live/dead cell staining, and the results are shown in Figure 7. Calcein–AM is a membrane-permeable green fluorescent dye that indicates live cells. PI is a membrane-impermeable red dye, which will only stain dead cells and indicates dead cells. Figure 7a shows fluorescence microscope images of MC3T3-E1 cells after co-culture with extracts of different fibrous membranes. Figure 7b is a statistical analysis of the cell number. It can be seen that the addition of ZIF-8 decreased the cell density in the PVA/ZIF-8 group, but there is no excessive increase in dead (red) cells. When Van and V@Z are added, the cell density returns to a level similar to that of the PVA group. The proportion of live cells and dead cells in each group is similar, which indicates that each group of fibrous membrane has good biocompatibility.

**Figure 8:**
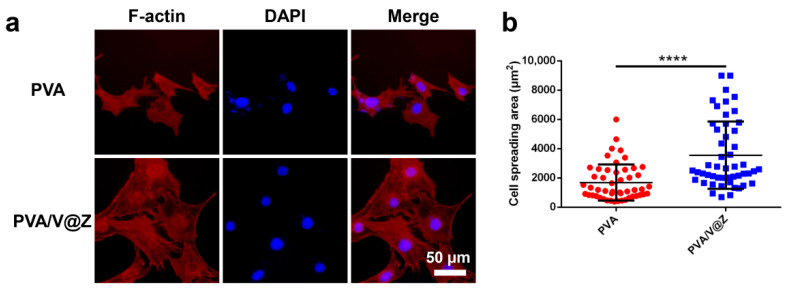
(**a**) Images of MC3T3-E1 cells stained with F-actin (red) and nucleus (blue) after being cultured on different fibrous membranes for 72 h: TRITC phalloidin: F-actin; DAPI: nuclei; Merge: merged channel of F-actin and DAPI; (**b**) Corresponding statistical results of cell spreading area. Mean values and standard deviations of 50 cells are presented. (**** *p* < 0.0001).

The description of the cytoskeleton can reflect the morphological changes of eukaryotic cells. Observing the spreading of cells can further explain the biocompatibility of materials. Figure 8a and Figure S4 show fluorescence microscope observation images of cells stained with TRITC phalloidin/DAPI. Phalloidin can specifically bind to F-actin in eukaryotic cells and stain the cytoplasm. DAPI can stain the nucleus. Their combination can show the distribution of cytoskeleton. In Figure 8a and Figure S4, it can be seen that similar results are shown between the groups, and MC3T3-E1 cells maintain good spreading. In the PVA/V@Z groups, the spreading area of a single cell increased. This is because the existence of particles in fibers can provide more adhesion sites for cells and promote its spreading area [22]. In addition, the Zn element released from V@Z can also promote the growth of cells. The area statistics of cells on PVA and PVA/V@Z membranes were carried out (Figure 8b). The average spreading area of cells on the PVA/V@Z membrane is as high as 3644 μm^2^, which is significantly higher than that on the PVA membrane (1690 μm^2^). The microfilament structure in the cell has established a good contact and formed a complete network, which has prepared the structural basis for further cell proliferation.

## 3. Materials and Methods

### 3.1. Material

Zinc nitrate hexahydrate (Zn(NO_3_)_2_·6H_2_O) was purchased from Sigma-Aldrich (St. Louis, MO, USA). 2-Methimidazole was purchased from Rhawn (Shanghai, China). Van was purchased from Yuanye Bio-Technology Ltd. (Shanghai, China). PVA and phosphoric acid were purchased from Aladdin Reagent Co., Ltd. (Shanghai, China). Anhydrous methanol was purchased from Fengchuan Chemical Reagent Technology Co., Ltd. (Tianjin, China). Glutaraldehyde fixation solution was purchased from Beijing Leagene Biotechnology Co., Ltd. (Beijing, China).

### 3.2. Synthesis of ZIF-8 and V@Z Particles

ZIF-8 particles were synthesized following the synthesis method reported by Karakecili [11] et al. ZIF-8 particles were synthesized at room temperature. An aqueous solution containing Zn(NO_3_)_2_·6H_2_O (0.5 mol/L, 3 mL) and an aqueous solution containing 2-methimidazole (3.47 mol/L, 30 mL) were mixed and stirred vigorously for 30 min. The product was collected by centrifugal machine at 12,000 r/min for 20 min and washed with methanol four times. The product was dried in the fume hood overnight.

V@Z was also synthesized at room temperature. The Zn(NO_3_)_2_·6H_2_O (0.25 mol/L, 4 mL) aqueous solution containing Van (25 mg) was mixed with the 2-methimidazole (3.84 mol/L, 18 mL) aqueous solution under stirring. Then, the same process as the synthesis of ZIF-8 was carried out.

### 3.3. Preparation of the Electrospun Fibrous Membrane

The fibrous membranes were prepared by electrospinning. PVA was stirred at 90 °C with deionized water, forming PVA solution (9 wt % of water mass). Quantitative ZIF-8 or V@Z particles (10 wt % of PVA mass) were added to PVA solution, respectively. In previous studies, it was explored that the appropriate doping amount of ZIF-8 was 10 wt % of PVA (Figures S5 and S6). The mixed solution was stirred to fully disperse the particles in PVA solution. The spinning operation was carried out with the 5 mL syringe equipped with 19 G needle. The prepared membranes were named PVA, PVA/ZIF-8, and PVA/V@Z. In addition, the PVA/Van solution was prepared. The content of Van in PVA/Van was the same as that in PVA/V@Z, which was calculated by the drug-loading rate of V@Z. The voltage of 8 kV was applied, and the flow rate was 0.038 mm/min. The membranes were dried at room temperature and crosslinked for 15 min in an oven at 150 °C, so that the membrane would not dissolve in water and retain its fiber structure. The processed membranes were stored in the refrigerator at −18 °C for subsequent use.

### 3.4. Characterization of the Particles and Electrospun Fibrous Membranes

The morphology of particles and electrospun membranes was observed by SEM (JSM7100F, JEOL Ltd., Tokyo, Japan). The element distribution of the particles and electrospun fibers was explored by TEM (Talos F200S, FEI, Hillsboro, OR, USA). XRD analysis of ZIF-8 and V@Z particles was performed with an X-ray diffractometer (D8 Discover, Bruker, Karlsruhe, Germany) range of 5–40°. FTIR (TENSOR 27, Bruker, Karlsruhe, Germany) was used to explore whether the drug was successfully loaded in particles and membranes. The hydrophilicity of the membrane was tested by water contact angle instrument (JC 2000DM, Shanghai Zhongchen Digital Technic Apparatus Co., Ltd., Shanghai, China).

### 3.5. Drug-Loading Rate (DL) of V@Z Particles

The DL of V@Z particles was measured by ultraviolet-visible spectrophotometer (UV-Vis) (UV100-star2, InsMark, Shanghai, China). Firstly, a series of Van solutions with different concentrations were prepared with deionized water to detect the absorption peak (Abs) at the wavelength of 280.2 nm. The standard curve of Van was gained. Quantitative V@Z particles were dispersed in deionized water, and an appropriate amount of phosphoric acid was added dropwise until the nanoparticles disappeared. At this time, the Abs value of the solution at the wavelength of 280.2 nm was measured, and the Van concentration in the solution was calculated according to the standard curve so as to obtain the corresponding weight. The DL of V@Z particles was calculated by the formula of DL (%) = (M_DN_/M_N_) × 100%, where M_DN_ represents the weight of Van in particles and M_N_ represents the weight of V@Z particles [35].

### 3.6. Water Absorption Rate of the Electrospun Fibrous Membrane

Water absorption study was performed by soaking the electrospun membrane (PVA, PVA/ZIF-8, PVA/Van and PVA/V@Z) of known weight (W_dry_) in deionized water at 37 °C. The membranes were removed from the water after soaking for 1 h. The wet weight (W_wet_) was measured after removing the surface water of the membrane. The water absorption rate (R_w_) was calculated by the formula of R_w_ (%) = (W_wet_ − W_dry_)/W_dry_ × 100%, where W_wet_ was the weight of the membrane after water absorption and W_dry_ was the weight of the dry membrane.

### 3.7. Van Release from Electrospun Fibrous Membrane

PVA/Van and PVA/V@Z membranes were, respectively, immersed in 4 mL PBS and maintained in a shaker at 100 r/min and 37 °C. In order to explore the pH effect of V@Z particles, two pH values of 6.5 and 7.4 were used for testing. At predetermined time points, the release medium (3 mL) was taken out to measure the absorbance of Van by UV-Vis spectrophotometry at 280.2 nm wavelength. Then, 3 mL of fresh PBS was added to maintain the same volume. The released amount of Van was determined by a standard curve.

### 3.8. Antibacterial Activity

*E. coli* CMCC(B)44102 and *S. aureus* ATCC6538 were used as experimental strains for an antibacterial test. Bacteria were cultured with LB liquid medium overnight under 37 °C in a shaker at 100 r/min. The experimental groups were set as PVA, PVA/ZIF-8, PVA/Van, and PVA/V@Z.

To test the bacteria-adhering profile on the surface, the membranes (diameter, 8 mm) were sterilized under UV for 1 h and then placed in a 48-well plate. The bacterial solution was diluted to about 10^7^ CFU/mL with PBS of pH 6.5 and 7.4, respectively. Each electrospun membrane was incubated with 500 µL bacterial solution under 37 °C for 12 h. Then, the membranes were gently rinsed twice with sterile PBS. The electrospun membranes were immersed with 2.5% glutaraldehyde for at least 4 h. Then, a series of alcohol solutions with different concentrations (30, 50, 70, 80, 85, 90, 95, and 100%) were successively used for gradient dehydration of the bacteria adhered to the membrane, and each concentration was dehydrated for 10 min. Finally, the sample was dried at room temperature.

To further investigate the antibacterial property in medium, the sterilized samples were placed in the Eppendorf tubes. The negative control group was bacterial solution. The bacterial solution was diluted to about 10^6^ CFU/mL with PBS of pH 6.5 and 7.4, respectively. Each electrospun membrane was incubated with 500 µL bacterial solution under 37 °C for 24 h. After the incubation period, the Eppendorf tubes were fully shaken with the vortex oscillator, and 100 μL bacterial suspensions were taken out from each tube for dilution. The suspension was continuously diluted to 1:10, 1:100, 1:1000 and 1:10000, and 100 μL under each gradient was spread onto plates. The coated plates were placed in the incubator at 37 °C for 15 h for colony counting.

### 3.9. Cell Culture and Bioactivity Characterizations

Cell culture: MC3T3-E1 cells were obtained from Orthopaedic Institute, Tianjin Medical University (Tianjin, China). The cells were cultured in basal medium (low-glucose DMEM with 10% FBS (Gibco, Thermo Fisher, Waltham, MA, USA), 1 ng/mL basic fibroblast growth factor and 1% penicillin/streptomycin). Cells were passaged using Trypsin after reaching 70–80% confluency.

Cytocompatibility: The cytocompatibility of the membrane was explored by using extraction solution. The membranes (diameter, 6 mm) were sterilized under UV for 1 h and then rinsed with PBS for 3 times. Then, we put samples in DMEM medium according to the extraction ratio of 1 cm^2^/mL (material surface area/extraction medium) to prepare the material extraction solutions. Each group was set with 5 parallel samples. The negative control group was DMEM culture medium.

MC3T3-E1 cells were seeded in 96-well plates at a density of 7 × 10^3^ cells/well and cultured at 37 °C, 5% CO_2_ for 24 h. Then, the basal medium was exchanged with material extraction solutions for another 24 h incubation. After the extract was co-cultured with the cells for 24 h, CCK-8 solution (Solarbio, Beijing, China) was added according to 1:10 of the volume of the culture medium. Then, the plate was placed in the incubator for 2 h. The absorbance at 450 nm for each well was measured by the microplate reader (Wellscan MK3, Labsystems Dragon, Finland).

Cell morphology: In addition, in order to further explore the cytotoxicity of the material, the time of cell extract was extended to 72 h for cell live/dead staining. The specific experimental operation was the same as the above, except that the extraction time was extended to 72 h. After the extract was co-cultured with the cells for 24 h, the cells were stained with a Calcein AM/PI double-stain kit (Solarbio, Beijing, China) to explore the cell-spreading behavior.

In order to further observe the regulatory effect of the materials’ physical structure on cell behavior, the method of directly seeding cells on materials was adopted. The membranes were rinsed with PBS for 3 times in a 96-well plate. The cells were seeded on the membrane at the density of 3 × 10^3^ cells/well and cultured for 72 h. TRITC phalloidin (Solarbio, Beijing, China) and DAPI (US Everbright, Suzhou, China) dye were used to stain cells. Then, they were observed via inverted fluorescence microscope (XD, SOPTOP, Shanghai, China).

### 3.10. Statistic Analysis

The data were expressed as means ± SD. One-way ANOVA was used to analyze the cell data in Figure 6, Figure 7 and Figure S6 and the bacterial data in Figure S5. Two-way ANOVA was used to analyze the bacterial data in Figure 5. A t-test was used to analyze the cell data in Figure 8. The *p* < 0.05 was considered statistically significant (* *p* < 0.05, ** *p* < 0.01, *** *p* < 0.001, **** *p* < 0.0001).

## 4. Conclusions

V@Z nanoparticles were successfully synthesized by a facile one-step method at room temperature. These nanoparticles were encapsulated in PVA fibers by electrospinning, and the electrospun fibrous membrane with structure-like ECM was prepared. The PVA/V@Z membrane has the characteristic of pH-dependent release, which can accelerate the drug release in the micro acid part of bacterial infection, and realize the anti-adhesion effect and slow release sterilization. The membrane has good biocompatibility and can increase the spreading area of a single MC3T3-E1 cell. The membrane is considered to be an ideal bone substitute and antimicrobial carrier for the treatment of serious bone infections such as osteomyelitis.

## Data Availability

The authors declare that all data supporting the findings are available within the paper or are available from the authors upon request.

## Supplementary Materials

The following supporting information can be downloaded at: https://www.mdpi.com/article/10.3390/ijms23105629/s1.
