# Supplementary material for: Antibacterial Vancomycin@ZIF-8 Loaded PVA Nanofiber Membrane for Infected Bone Repair
Source: Int J Mol Sci. 2022 May 18;23(10):5629. doi: 10.3390/ijms23105629 (PMC9145349; doi:10.3390/ijms23105629)
Supplement: Supplementary file 1 [file ijms-23-05629-s001.zip › ijms-1708193-supplementary.pdf]

International Journal of *Molecular Sciences*

MDPI

Supplementary Materials

Antibacterial Vancomycin@ZIF-8 Loaded PVA Nanofiber Membrane for Infected Bone Repair

Yunbo Zhao[1], Hongshui Wang[1], Xianrui Zou[1], Donghui Wang[1], Ying Fan[1], Xiaoyan Zhao[2], Mingjun Li[3,*], Lei Yang[3] and Chunyong Liang[1,4,*]

1 Tianjin Key Laboratory of Materials Laminating Fabrication and Interface Control Technology, School of Materials Science and Engineering, Hebei University of Technology, Tianjin 300130, China; 15733153963@163.com (Y.Z.); kingflood@hebut.edu.cn (H.W.); zouxianrui@163.com (X.Z.); wdh_81@163.com (D.W.); fanying_99@yahoo.com (Y.F.)
2 School of Civil and Transportation Engineering, Hebei University of Technology, Tianjin 300130, China; zhaoxiaoyan@hebut.edu.cn
3 Center for Health Science and Engineering, School of Health Sciences and Biomedical Engineering, Hebei University of Technology, Tianjin 300130, China; ylei@hebut.edu.cn
4 Fujian Provincial Key Laboratory for Advanced Micro-Nano Photonics Technology and Devices, Research Center for Photonics Technology, Quanzhou Normal University, Quanzhou 362000, China
* Correspondence: mjli@hebut.edu.cn (M.L.); liangchunyong@hebut.edu.cn (C.L.)

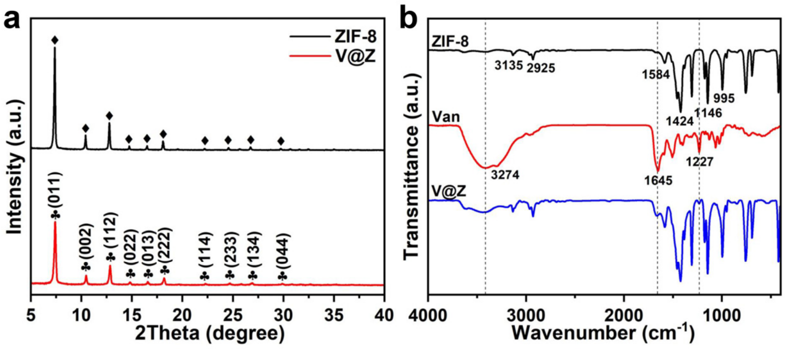

Figure S1. XRD patterns of ZIF-8 and V@Z(a); FTIR of ZIF-8, Van and V@Z(b).

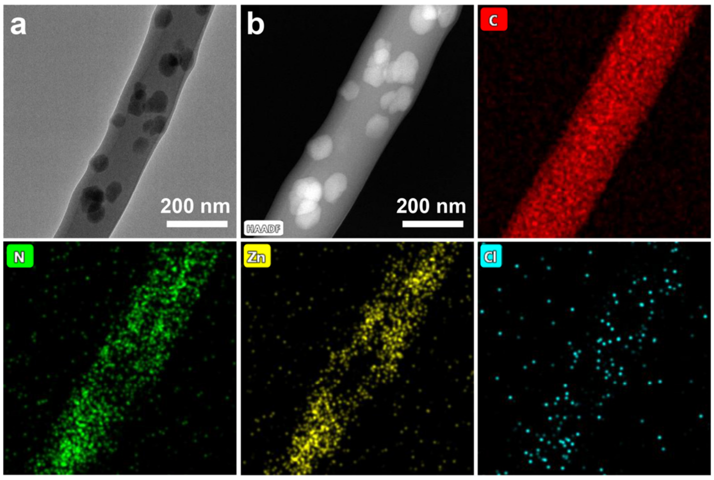

Figure S2. TEM image (a), and elemental mapping (b) of PVA/V@Z fiber.

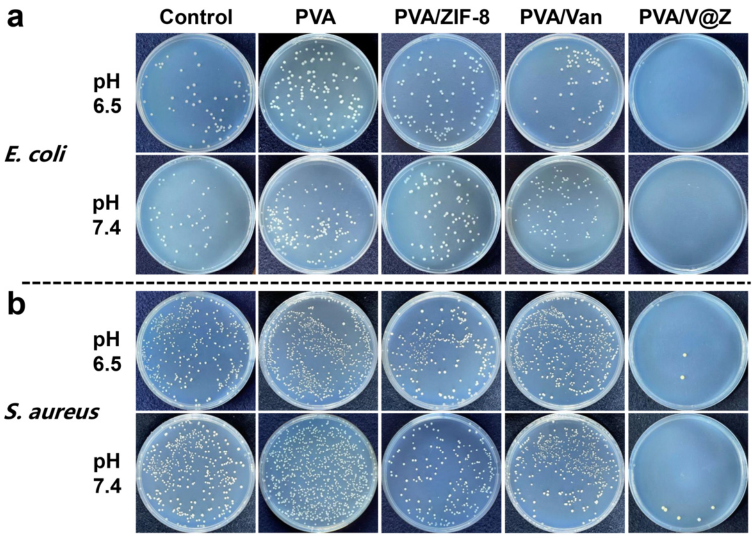

Figure S3. Representative images of agar plates after 24 h co-culture with different membranes (diluted 10,000 times): *E. coli* (a) and *S. aureus* (b).

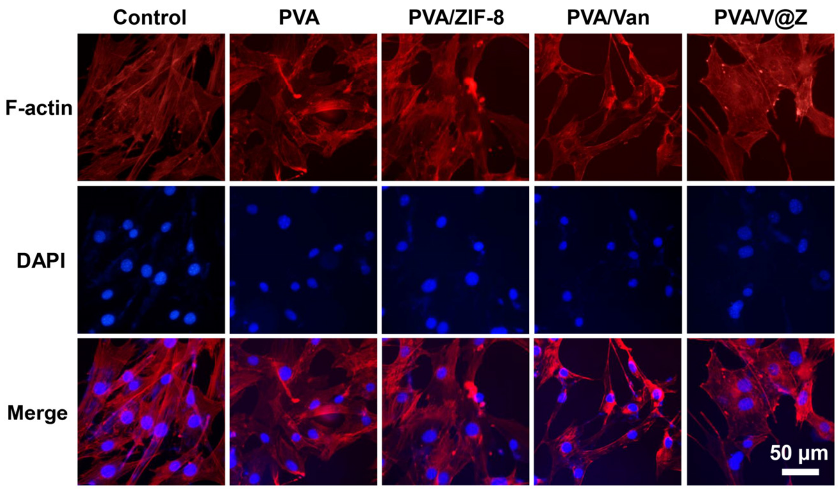

Figure S4. Images of MC3T3-E1 stained with F-actin (red) and nucleus (blue) after cultured on different fibrous membranes for 72 h: TRITC phalloidin: F-actin; DAPI: nuclei; Merge: merged channel of F-actin and DAPI.

The suitable doping concentration of ZIF-8 particles in PVA was explored. Three kinds of PVA/ZIF-8 fibrous membranes with different ZIF-8 content were prepared, which were 5 wt%, 10 wt% and 15 wt% of PVA mass. Firstly, the antibacterial differences between membranes were explored. Membranes were co-cultured with 500 μL 10^6 CFU/mL bacterial solution for 24 h, and the experimental operation is the same as that in the article. The experimental conditions with pH 7.4 were only set here. Figure S5a and c are the representative images of agar plates after diluting the original bacterial solution 10000 times. Figure S5b, d are the corresponding data statistics. The antibacterial efficiency of the membranes increased with the increase of ZIF-8 content. The antibacterial efficiency of the 10 wt% membrane against *E. coli* is about 38%, and against *S. aureus* is about 76%.

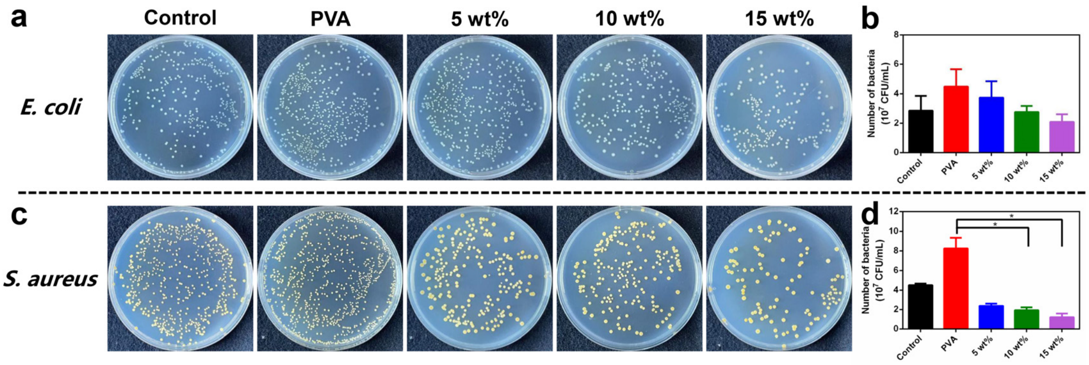

Figure S5. Representative images of agar plates and corresponding data statistics after 24 h co-culture with different membranes (diluted 10,000 times): *E. coli* (a, b) and *S. aureus* (c, d) (n = 3). (* $p < 0.05$)

Secondly, the cell viability after co-culture with different fibrous membrane extracts for 24 h was tested via CCK-8 assay kit, and the experimental operation is the same as that in the article. As shown in Figure S6, when the doping concentration of ZIF-8 reaches 15 wt%, the membrane extract shows a certain cytotoxicity. Therefore, the doping concentration of ZIF-8 was determined to be 10 wt% for subsequent experimental operations. The membrane at this concentration has certain antibacterial properties and good biocompatibility.

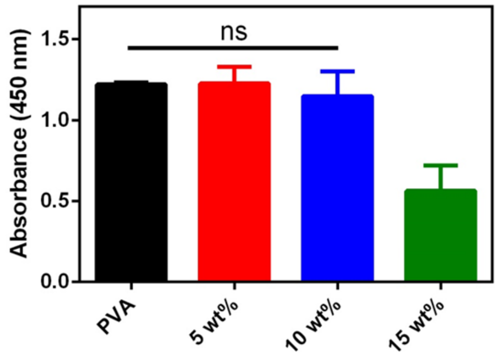

Figure S6. CCK-8 results after 24 h co-culture of different membrane extracts and MC3T3-E1 cells (n = 5). (ns, not significant)
